# Hypoxic Exercise Alleviates Diabetic Skeletal Myopathy in Zebrafish in Association with Hif1a/Foxo3a Signaling and Mitochondrial Quality Control

**DOI:** 10.3390/ijms27188034

**Published:** 2026-09-09

**Authors:** Haoming Li, Zhanglin Chen, Qinghua Deng, Yunyi Zou, Shuaiwang Huang, Bihan Wang, Yelin Zeng, Lan Zheng, Changfa Tang, Zuoqiong Zhou, Yishan Chen, Xiyang Peng

**Affiliations:** 1 College of Physical Education, Hunan Normal University, Changsha 410081, China; lihaoming@hunnu.edu.cn (H.L.); zhanglinchen@hunnu.edu.cn (Z.C.); 202270153046@hunnu.edu.cn (Q.D.); 202020151346@hunnu.edu.cn (Y.Z.); 202320152962@hunnu.edu.cn (S.H.); wbhxhzz@hunnu.edu.cn (B.W.); 202230171048@hunnu.edu.cn (Y.Z.); lanzheng@hunnu.edu.cn (L.Z.); changfatang@hunnu.edu.cn (C.T.); zhouzuoqiong@hunnu.edu.cn (Z.Z.); 2 Hunan Center for Public Sports Service Research, Hunan Normal University, Changsha 410081, China; 3 Key Laboratory of Physical Fitness and Exercise Rehabilitation of Hunan Province, Hunan Normal University, Changsha 410081, China

**Keywords:** diabetic myopathy, hypoxic exercise, mitochondrial quality control, hif1a, zebrafish

## Abstract

Type 2 diabetes mellitus (T2DM) is characterized by skeletal muscle insulin resistance, which contributes to systemic metabolic dysregulation and diabetic myopathy (DM), a complication involving the loss of muscle mass and contractile function. Although hypoxic exercise has demonstrated metabolic benefits in chronic diseases, its efficacy against DM remains unclear. We hypothesized that hypoxic exercise would be more effective than normoxic exercise in ameliorating diabetic myopathy-associated skeletal muscle abnormalities. Accordingly, adult zebrafish underwent an 8-week high-glucose intervention combining high-glucose feeding and glucose immersion to establish a T2DM-associated diabetic myopathy model and were subsequently subjected to 5 weeks of normoxic or hypoxic exercise (28.8 cm/s, corresponding to 70% of critical swimming speed (Ucrit) determined under normoxic conditions, 2 h/day, 5 days/week) to compare their effects on skeletal muscle outcomes. Hypoxic exercise produced greater improvements in glucose tolerance and locomotor performance than normoxic exercise, while the effects on skeletal muscle morphological and atrophy-related indices varied across outcomes. Both normoxic exercise and intermittent hypoxic exposure altered the abundance of mitochondrial quality-control-related proteins in the skeletal muscle of zebrafish with diabetic myopathy, whereas hypoxic exercise produced more pronounced changes in several of these proteins. Moreover, hypoxic exercise more markedly increased the abundance of mitochondrial respiratory-chain proteins than aerobic exercise or intermittent hypoxic exposure alone. Marked reactive oxygen species (ROS) accumulation was observed in the skeletal muscle of zebrafish with diabetic myopathy. Both aerobic and hypoxic exercise effectively reduced excessive ROS production, with hypoxic exercise producing the greater effect. Analysis of Foxo3a and its phosphorylation showed that p-Foxo3a expression decreased most prominently in the T2DM hypoxia exercise (DHE) group, indicating that hypoxic exercise is associated with modulation of Hif1a/Foxo3a-related signaling. Hypoxic exercise was associated with greater Hif1a protein abundance and lower Foxo3a Ser253 phosphorylation than normoxic exercise, accompanied by improved mitochondrial quality control and reduced ROS accumulation. In conclusion, hypoxic exercise ameliorated diabetic skeletal myopathy and was accompanied by modulation of Hif1a/Foxo3a-related signaling, more favorable mitochondrial morphology, altered abundance of mitochondrial quality-control-related and respiratory-chain proteins, and reduced dihydroethidium-detectable oxidative signals.

## 1. Introduction

Type 2 diabetes mellitus (T2DM) is a prevalent metabolic disorder characterized by normal or excessive insulin secretion from pancreatic β-cells, yet diminished biological efficacy of insulin, leading to insulin resistance (IR) [1]. This condition further triggers endocrine and metabolic disorders, giving rise to a spectrum of severe complications, including blindness, renal failure, cardiovascular diseases, and skeletal muscle dysfunction [2]. Diabetic myopathy represents a skeletal muscle complication associated with T2DM and hallmarked by hyperglycemia [3]. Its primary manifestations include reduced skeletal muscle mass and impaired muscle function, which compromise patients’ quality of life and elevate mortality [4].

As a vital motor organ in the human body, skeletal muscle not only undertakes body movement and postural maintenance but also plays a pivotal role in metabolic processes, particularly exerting an irreplaceable function in glucose metabolism and energy homeostasis. Skeletal muscle can regulate blood glucose concentrations and promote glucose uptake and utilization through both insulin-dependent and insulin-independent pathways [5]. Nevertheless, a reciprocal interaction exists between disturbed glucose metabolism and skeletal muscle function. Studies have demonstrated that both type 1 and type 2 diabetes are characterized by the progressive decline in skeletal muscle mass and function, defined as diabetic myopathy [6,7]. Under this pathological condition, a series of structural, functional, and metabolic alterations occur in skeletal muscle, specifically manifested as myofiber atrophy [8], altered secretion of myokines [9], impaired mitochondrial structure and bioenergetics [10], as well as fiber-type switching and reduced oxidase activity [11]. Collectively, these changes ultimately lead to decreased muscle strength [6], compromised functional capacity, and even elevated mortality in patients [12].

As the intracellular “powerhouse”, mitochondria exert a direct impact on the metabolic capacity of skeletal muscle. Mitochondria are not only responsible for energy production but also participate in fatty acid oxidation. Impaired mitochondrial function results in reduced fatty acid oxidation capacity, thereby triggering a cascade of abnormalities, including lipid accumulation in skeletal muscle [13]. Mitochondria play a pivotal role in modulating metabolic pathways and maintaining tissue energy homeostasis, and diabetes is closely associated with compromised mitochondrial function, manifested specifically by decreased mitochondrial content [14], impaired lipid oxidation [15], and excessive generation of reactive oxygen species (ROS) [16]. Recent human evidence further indicates that T2DM is accompanied by altered mitochondrial dynamics and impaired mitochondrial quality-control signaling in skeletal muscle, linking abnormalities in mitochondrial quality control with reduced insulin sensitivity [17]. Furthermore, diabetes may suppress adequate mitophagy, leading to the accumulation of damaged mitochondria in skeletal muscle and subsequent disruption of energy metabolism [18]. Consistent with this, relevant animal studies have confirmed the presence of mitochondrial dysfunction in diabetic and obese mice, characterized by reduced mitochondrial DNA copy number, increased disorganized cristae, and decreased citrate synthase activity [19]. Excessive ROS derived from dysfunctional mitochondria damages cellular components, activates stress-sensitive signaling pathways, and further impairs insulin action [20,21]. Accumulating evidence indicates that FoxO (forkhead box class O) transcription factors exert multifaceted roles in the regulation and dysregulation of autophagy [22], among which Foxo3a modulates mitophagy through phosphorylation and nuclear translocation [23].

Hypoxic exercise, an intervention combining hypoxic exposure (passive) and exercise training (active), reduces oxygen supply to body tissues and lowers arterial oxygen saturation [24]. During an exercise training program, continuous or intermittent hypoxic stimuli are applied, utilizing the specific biological effects induced by artificially simulated or naturally occurring high-altitude hypoxia, in combination with exercise training to enhance systemic hypoxic stress. This strategy mobilizes the body’s functional reserve and triggers a series of anti-hypoxic physiological responses and adaptations conducive to improving exercise performance [25]. Over the past decade, intermittent hypoxic exercise during physical training in individuals with obesity has gained considerable attention [26]. Notably, a single session of aerobic exercise performed under moderate hypoxic conditions (approximately 3000 m altitude) significantly improves glucose tolerance and insulin sensitivity in obese patients with type 2 diabetes mellitus [27,28]. More recent studies have further shown that exercise performed under moderate hypoxia can improve post-exercise glucose and insulin responses, while repeated intermittent hypoxia with or without aerobic exercise can enhance skeletal muscle glucose utilization in experimental T2D [29]. Since participants are exposed to relatively safe moderate hypoxia (1800–3000 m), additional exercise augments the overall metabolic stress elicited by hypoxia, thereby achieving therapeutic effects that are difficult to attain under normoxic conditions [30].

Hypoxic responses involve a wide spectrum of biochemical and molecular processes, among which hypoxia-inducible factor (HIF) serves as the primary transcriptional regulator governing cellular adaptation to hypoxia [31], whose activity is predominantly modulated by oxygen availability [32]. In zebrafish, Hif1a-related hypoxic responses involve duplicated paralogs, including hif1aa and hif1ab. Because the present study assessed Hif1a protein abundance by Western blotting, the detected signal was interpreted at the protein level. Hif1a protein abundance is responsive to hypoxic exercise and may contribute to beneficial metabolic responses, including glucose homeostasis and insulin sensitivity [33]. Accumulating evidence has demonstrated that under experimental hypoxic conditions in both cultured cells and hypoxia-exposed mice [34], Hif1a-related signaling has been implicated in the regulation of Foxo3a activity under hypoxic conditions. Activated Foxo3a translocates into the nucleus, regulates the expression of genes associated with mitophagy, facilitates the removal of damaged mitochondria, and preserves mitochondrial functional integrity. Nevertheless, the relationship between Hif1a/Foxo3a signaling and the beneficial effects of hypoxic exercise on mitochondrial alterations and skeletal muscle performance in diabetic myopathy remains incompletely understood. We hypothesized that, in zebrafish with diabetic myopathy, hypoxic exercise would induce greater improvements in glucose tolerance, skeletal muscle morphology, and locomotor performance than either normoxic exercise or hypoxic exposure alone, and that these benefits would be associated with modulation of Hif1a/Foxo3a-related signaling and altered abundance of mitochondrial quality-control-related proteins. Building on our previously established zebrafish model of diabetic myopathy [35], the present study extends our earlier work from model characterization to intervention-based investigation. Specifically, we compared normoxic exercise, hypoxic exposure, and hypoxic exercise within the same diabetic background to determine whether combined hypoxic exercise produces greater improvements in skeletal muscle phenotype and mitochondrial quality-control-related responses. We further examined whether these changes are accompanied by modulation of Hif1a/Foxo3a-related signaling.

## 2. Results

### 2.1. Hypoxic Exercise Significantly Improves Skeletal Muscle Glucose Tolerance and Lipid Metabolism in Zebrafish with Diabetic Myopathy

The four intervention groups were defined as T2DM normoxia control (DC), T2DM normoxia exercise (DE), T2DM hypoxia control (DHC), and T2DM hypoxia exercise (DHE).

**Figure 1:**
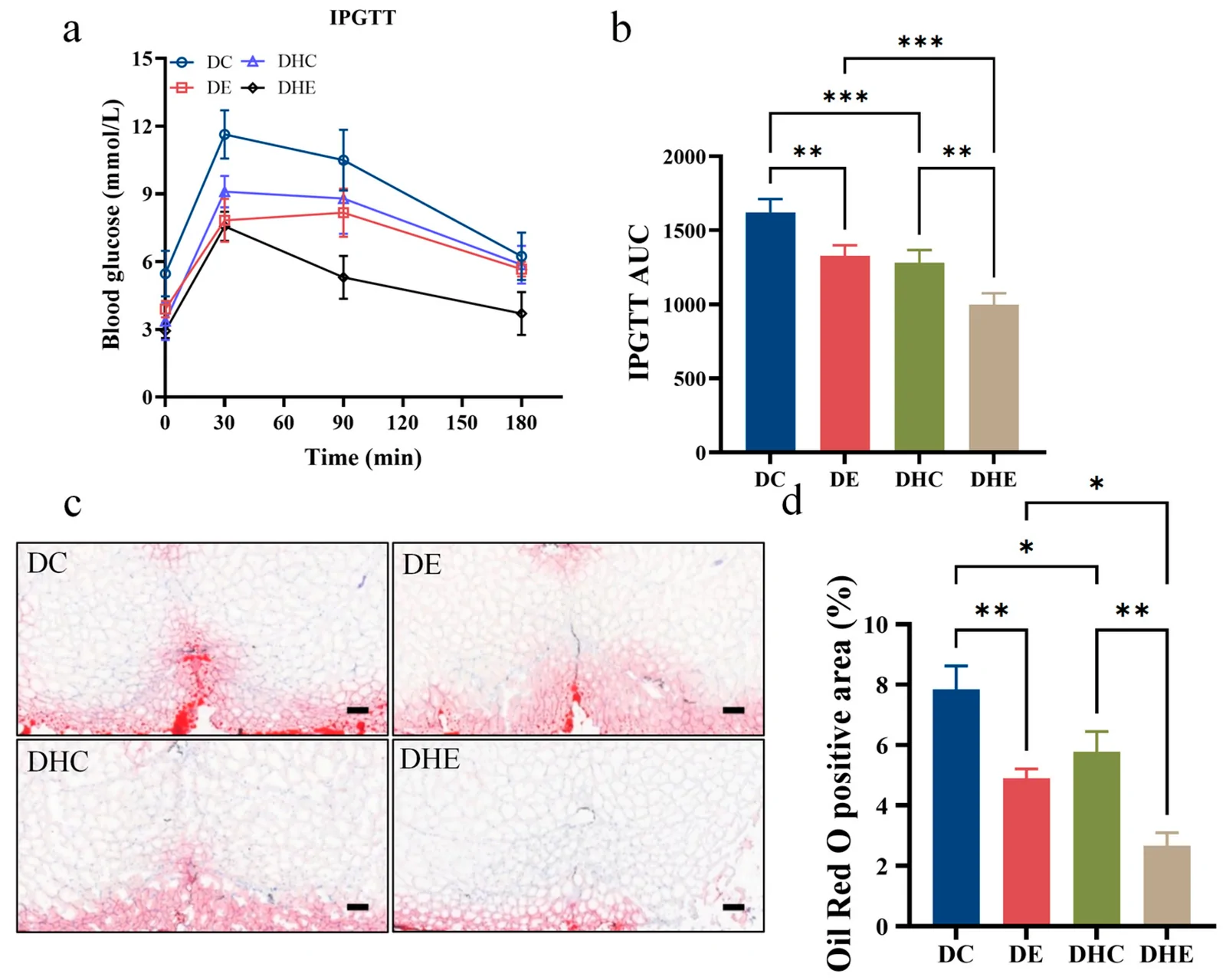
Effects of hypoxic exercise on glucose tolerance and lipid accumulation in zebrafish. (**a**) Intraperitoneal glucose tolerance test (IPGTT) curves in each group (*n* = 6 biological replicates per group); (**b**) area under the curve (AUC) of the IPGTT (*n* = 6 biological replicates per group); (**c**) representative Oil Red O staining images of zebrafish skeletal muscle. Scale bar = 50 μm; (**d**) quantitative analysis of the Oil Red O-positive area (*n* = 3 biological replicates per group). For Oil Red O quantification, three non-adjacent sections were analyzed per zebrafish, with six randomly selected, non-overlapping fields of view analyzed per section; measurements were averaged to obtain one value per animal. Data are presented as mean ± SD. Statistical analysis was performed using two-way ANOVA followed by Tukey’s multiple comparisons test. Statistical comparisons were performed among the DC, T2DM normoxia control; DE, T2DM normoxia exercise; DHC, T2DM hypoxia control; DHE, T2DM hypoxia exercise; SD, standard deviation; ANOVA, analysis of variance; * *p* < 0.05, ** *p* < 0.01, *** *p* < 0.001.

The intraperitoneal glucose tolerance test (IPGTT) serves as a key approach for diagnosing type 2 diabetes mellitus and evaluating systemic glucose metabolism. In the present study, zebrafish with diabetic myopathy were subjected to 5 weeks of exercise intervention under different oxygen concentrations, followed by IPGTT assessment using an intraperitoneal glucose dose of 0.5 mg/g body weight. Compared with the DC group, glucose tolerance was significantly improved in both the DE and DHC groups, with a more pronounced improvement observed in the DHE group (Figure 1a,b). Subsequently, Oil Red O staining was performed to investigate lipid accumulation in zebrafish skeletal muscle. The results demonstrated that lipid deposition was markedly alleviated in the DE and DHC groups compared with the DC group. Meanwhile, the extent of skeletal muscle lipid accumulation in the DHE group was significantly lower than that in the DE and DHC groups (Figure 1c,d). These findings indicate that both exercise and hypoxic exposure contribute to improvements in glucose tolerance and reductions in skeletal muscle lipid accumulation, with the combined DHE condition showing the most pronounced changes among the four diabetic groups.

Before the exercise intervention, metabolic assessment confirmed the diabetic phenotype of the T2DM zebrafish, as evidenced by elevated fasting blood glucose, impaired glucose tolerance, increased IPGTT area under the curve (AUC), and elevated glycated hemoglobin A1c (HbA1c) levels compared with normal control (NC) zebrafish (Supplementary Figure S2). Terminal fasting blood glucose and body weight following the 5-week intervention are presented in Supplementary Figure S3.

### 2.2. Effects of Hypoxic Exercise on Skeletal Muscle Morphology and Atrophy-Related Protein Abundance in Zebrafish with Diabetic Myopathy

**Figure 2:**
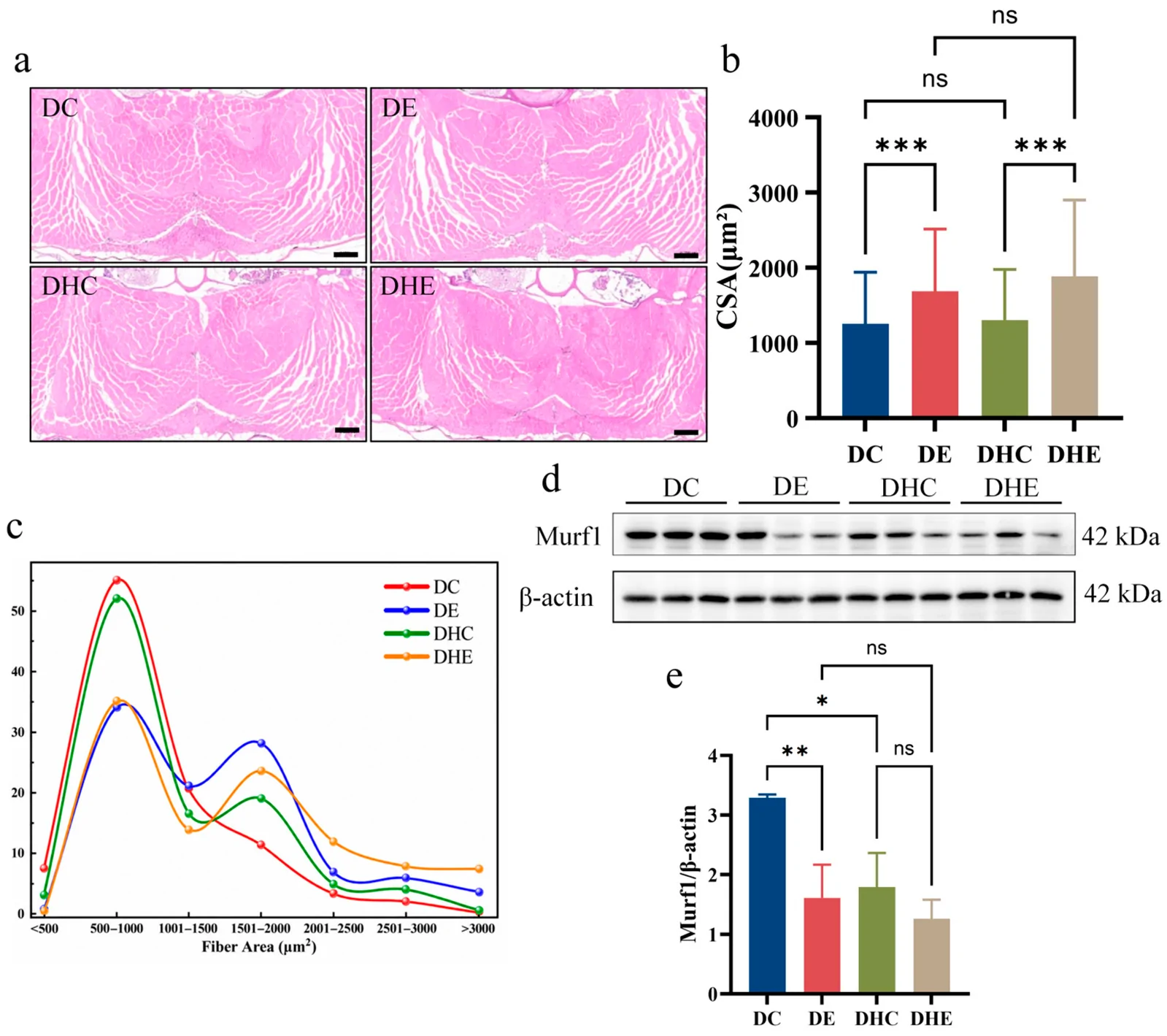
Effects of hypoxic exercise on skeletal muscle morphology and atrophy-related protein abundance in zebrafish with diabetic myopathy. (**a**) Representative hematoxylin and eosin (HE) staining images of zebrafish skeletal muscle. Scale bar = 100 μm; (**b**) quantitative analysis of skeletal muscle cross-sectional area (CSA); (**c**) distribution of skeletal muscle fiber CSA; (**d**) representative Western blot images of Trim63 and β-actin; (**e**) quantitative analysis of Trim63 protein abundance. For CSA quantification and fiber-size distribution analysis, *n* = 3 biological replicates per group. Three non-adjacent sections were analyzed per zebrafish, with six randomly selected, non-overlapping fields of view analyzed per section. Measurements from multiple sections and fields within each zebrafish were averaged to obtain one value per animal. For Western blot analysis, *n* = 3 biological replicates per group. Data are presented as mean ± SD. Statistical analysis was performed using two-way ANOVA followed by Tukey’s multiple comparisons test. Statistical comparisons were performed among the DC, DE, DHC, and DHE groups. ns, not significant; * *p* < 0.05, ** *p* < 0.01, *** *p* < 0.001.

Statistical analysis of the cross-sectional area (CSA) of the caudal muscle in each group revealed that compared with the DC group, the skeletal muscle CSA was significantly increased in the DE group, while no significant difference was observed between the DHC and DC groups. However, the CSA of skeletal muscle fibers in the DHE group was significantly larger than that in the DHC group (Figure 2a,b). Furthermore, statistical analysis of the CSA of individual skeletal muscle fibers showed that the fiber areas in the DC and DHC groups were mainly distributed in the range of less than 1500 μm^2^, whereas a higher proportion of fibers in the DE and DHE groups had a CSA greater than 2000 μm^2^ (Figure 2c). These results indicate that normoxic exercise increased skeletal muscle CSA relative to the DC group, whereas the DHE group showed a larger CSA than the DHC group. Subsequently, we detected the expression levels of marker proteins related to skeletal muscle atrophy (Figure 2d). The results showed that compared with the DC group, the expression level of Trim63 (a marker associated with skeletal muscle atrophy) was significantly decreased in both the DE and DHC groups, and there was no significant difference in Trim63 expression between the DHE group and the DE/DHC groups (Figure 2e).

### 2.3. Hypoxic Exercise Enhances Motor Capacity in Zebrafish with Diabetic Myopathy

**Figure 3:**
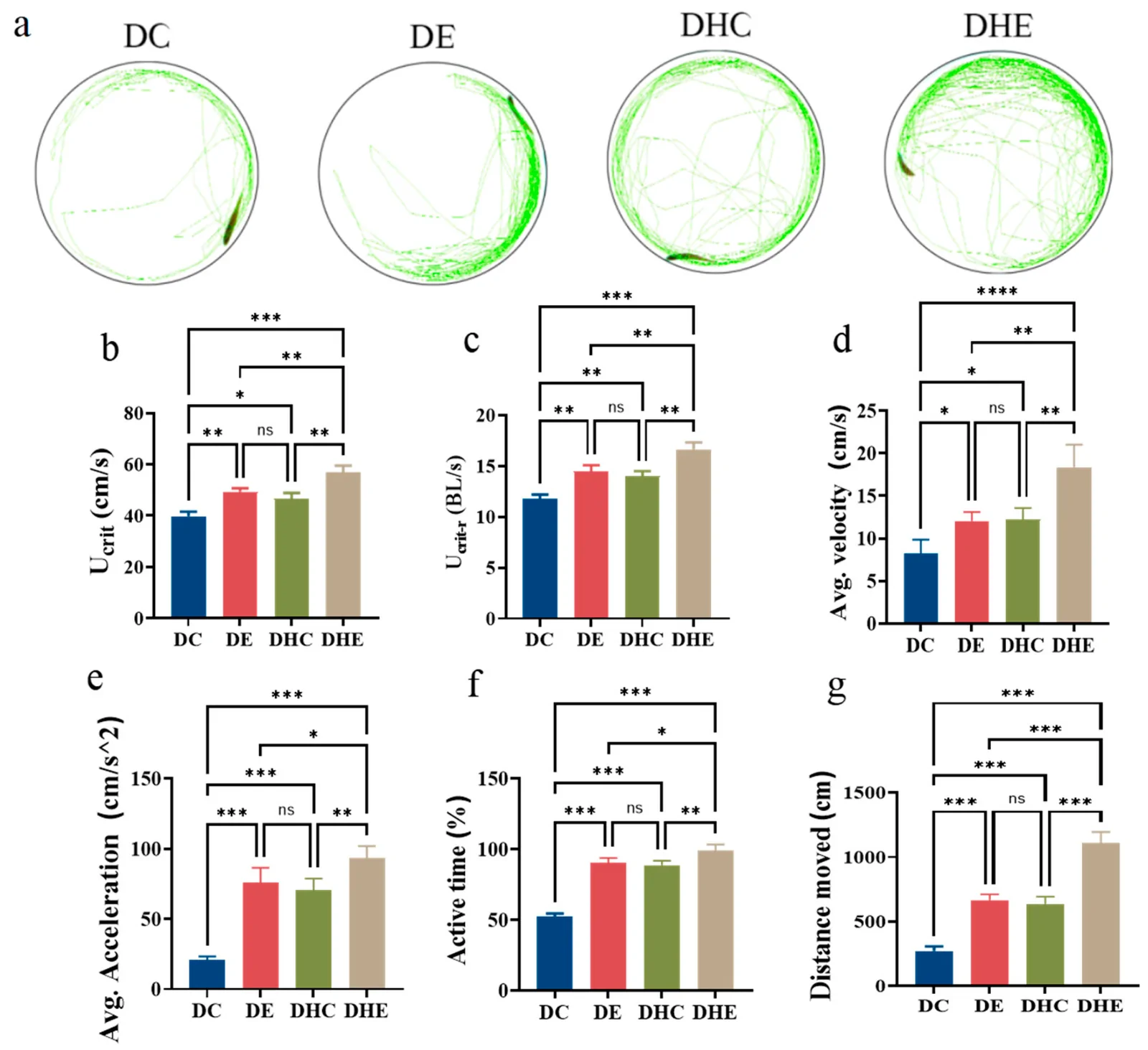
Effects of hypoxic exercise on locomotor performance in zebrafish with diabetic myopathy. (**a**) Representative traces from the open-field test; (**b**) critical swimming speed (Ucrit); (**c**) relative critical swimming speed (Ucrit-r); (**d**) average swimming velocity; (**e**) average swimming acceleration; (**f**) percentage of movement time; (**g**) total distance traveled. *n* = 6 biological replicates per group. Data are presented as mean ± SD. Statistical comparisons were performed among the DC, DE, DHC, and DHE groups. Statistical analysis was performed using two-way ANOVA followed by Tukey’s multiple comparisons test. ns, not significant; * *p* < 0.05, ** *p* < 0.01, *** *p* < 0.001, **** *p* < 0.0001.

After completion of the exercise intervention, to investigate whether motor capacity was altered, critical swimming speed (Ucrit) measurements and an open-field test were performed on zebrafish in each group (Figure 3a). The results showed that compared with the DC group, both Ucrit and Ucrit-r were significantly increased in the DE and DHC groups, and the increases in the DHE group were more significant than those in the DE and DHC groups (Figure 3b,c). In addition, the results of the open-field test demonstrated that compared with the DC group, the average swimming velocity (Figure 3d), average swimming acceleration (Figure 3e), percentage of movement time (Figure 3f), and total distance traveled (Figure 3g) were significantly elevated in the DE and DHC groups. Moreover, the magnitude of elevation in the DHE group was significantly higher than that in the DE and DHC groups.

These results suggest that hypoxic exercise exerts a more pronounced effect on enhancing the motor capacity of zebrafish with diabetic myopathy.

### 2.4. Hypoxic Exercise Improves Mitochondrial Morphology and Alters Mitochondrial Quality-Control-Related Protein Abundance in Skeletal Muscle of Zebrafish with Diabetic Myopathy

**Figure 4:**
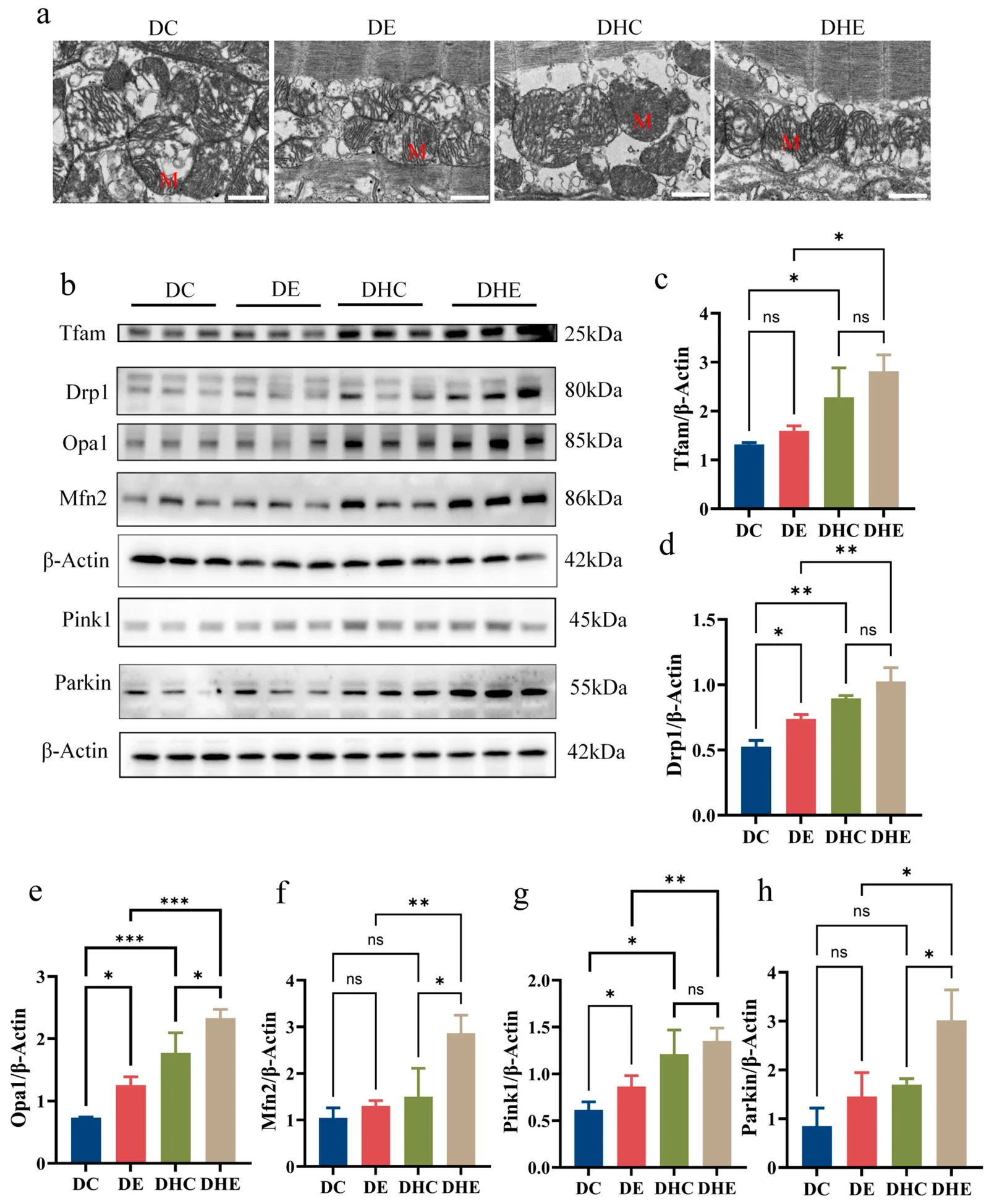
Effects of hypoxic exercise on mitochondrial morphology and mitochondrial quality-control-related protein abundance in skeletal muscle of zebrafish with diabetic myopathy. (**a**) Representative transmission electron micrographs of mitochondria in zebrafish skeletal muscle. M, mitochondria. Scale bar = 1 μm; (**b**) representative Western blot images of mitochondrial quality-control-related proteins; (**c**–**h**) quantitative analyses of Tfam, Drp1, Opa1, Mfn2, Pink1, and Parkin protein abundance. *n* = 3 biological replicates per group. For Tfam quantification, only the band corresponding to the expected molecular weight was included in densitometric analysis, whereas additional nonspecific bands were excluded. Data are presented as mean ± SD. Statistical analysis was performed using two-way ANOVA followed by Tukey’s multiple comparisons test. Statistical comparisons were performed among the DC, DE, DHC, and DHE groups. ns, not significant; * *p* < 0.05, ** *p* < 0.01, *** *p* < 0.001.

Mitochondria play a crucial role in cellular growth, metabolism, and synthesis, and the development of diabetic myopathy (DM) is also closely associated with mitochondrial dysfunction. First, transmission electron microscopy (TEM) analysis of skeletal muscle from zebrafish in each group revealed that mitochondria in the DC group exhibited vacuolation and cristae loss, while these abnormalities were alleviated in the DE, DHC, and DHE groups. Flameng’s score was used for semi-quantitative evaluation of mitochondrial morphology (Figure 4a). To further characterize mitochondrial quality-control-related molecular changes in the skeletal muscle of zebrafish with diabetic myopathy, we quantitatively compared the abundance of proteins involved in mitochondrial biogenesis, fission, fusion, and mitophagy, including Tfam, Drp1, Opa1, Mfn2, Pink1, and Parkin. Pink1 and Parkin are key regulators of ubiquitin-dependent mitophagy. Pink1 accumulates on damaged mitochondria and recruits Parkin to the mitochondrial outer membrane, thereby promoting the selective clearance of dysfunctional mitochondria. Compared with the DC group, the protein expression levels of Drp1 and Opa1 were significantly upregulated in the DE group; similarly, the expression levels of Tfam, Drp1, Opa1, and Pink1 were significantly increased in the DHC group. However, compared with the DE group, the expression levels of Tfam, Drp1, Opa1, Mfn2, Pink1, and Parkin were significantly upregulated in the DHE group (Figure 4b–h).

These findings indicate that both aerobic exercise and hypoxic exposure altered the abundance of mitochondrial quality-control-related proteins in the skeletal muscle of zebrafish with diabetic myopathy, with more pronounced changes observed following hypoxic exercise.

### 2.5. Hypoxic Exercise Alters Mitochondrial Respiratory-Chain Protein Abundance in Skeletal Muscle of Zebrafish with Diabetic Myopathy

**Figure 5:**
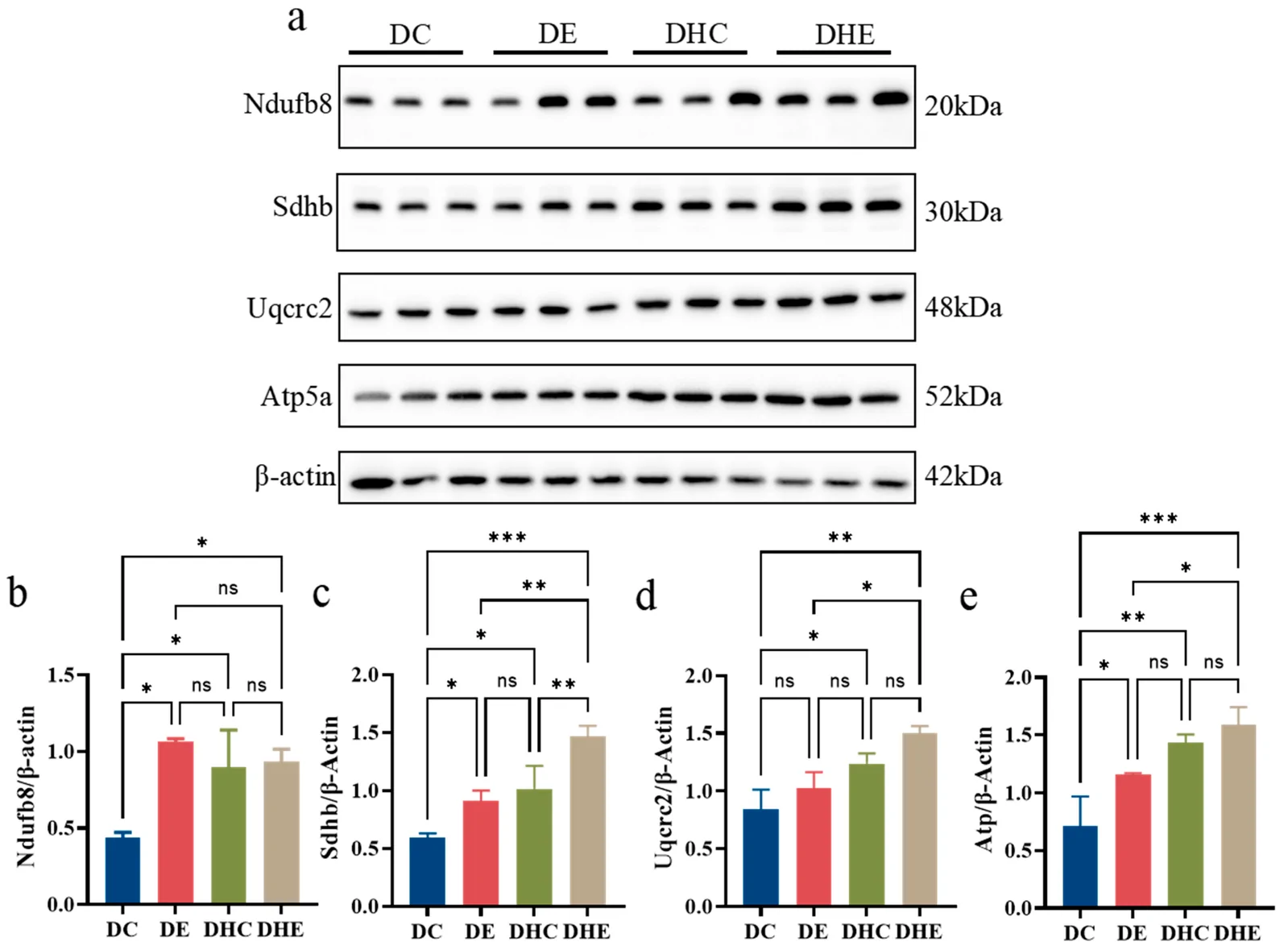
Effects of hypoxic exercise on mitochondrial respiratory-chain protein abundance in skeletal muscle of zebrafish with diabetic myopathy. (**a**) Representative Western blot images of mitochondrial respiratory-chain proteins; (**b**–**e**) quantitative analyses of Ndufb8, Sdhb, Uqcrc2, and Atp5a protein abundance. *n* = 3 biological replicates per group. Data are presented as mean ± SD. Statistical comparisons were performed among the DC, DE, DHC, and DHE groups. Statistical analysis was performed using two-way ANOVA followed by Tukey’s multiple comparisons test. ns, not significant; * *p* < 0.05, ** *p* < 0.01, *** *p* < 0.001.

Mitochondria are the primary site of aerobic respiration in cells, converting intracellular nutrients such as glucose, fatty acids, and amino acids into adenosine triphosphate (ATP) via oxidative phosphorylation. ATP synthesis mainly occurs during oxidative phosphorylation and represents the ultimate outcome of mitochondrial respiration [36]. We examined the expression levels of mitochondrial respiratory chain proteins, including complex I (Ndufb8), complex II (Sdhb), complex III (Uqcrc2), and complex V (Atp5a). Compared with the DC group, Ndufb8, Sdhb, and Atp5a protein abundance was significantly increased in the DE group. Similarly, Ndufb8, Sdhb, Uqcrc2, and Atp5a protein abundance was significantly increased in the DHC group relative to DC. Furthermore, compared with the DE group, the DHE group exhibited significantly higher abundance of Uqcrc2, Sdhb, and Atp5a (Figure 5a–e).

These results indicate that hypoxic exercise exerts a more pronounced effect on elevating the expression of key proteins in the mitochondrial respiratory chain in skeletal muscle of diabetic zebrafish compared with aerobic exercise.

### 2.6. Hypoxic Exercise Reduces Dihydroethidium-Detected Oxidative Signals in Skeletal Muscle of Zebrafish with Diabetic Myopathy

Reactive oxygen species (ROS) are oxides generated during mitochondrial ATP production via oxidative phosphorylation in the electron transport chain (ETC). Under physiological conditions, low levels of ROS act as signaling messengers. However, in diabetic myopathy, impaired mitochondrial function leads to excessive ROS accumulation, which exacerbates oxidative stress and accelerates mitochondrial damage.

**Figure 6:**
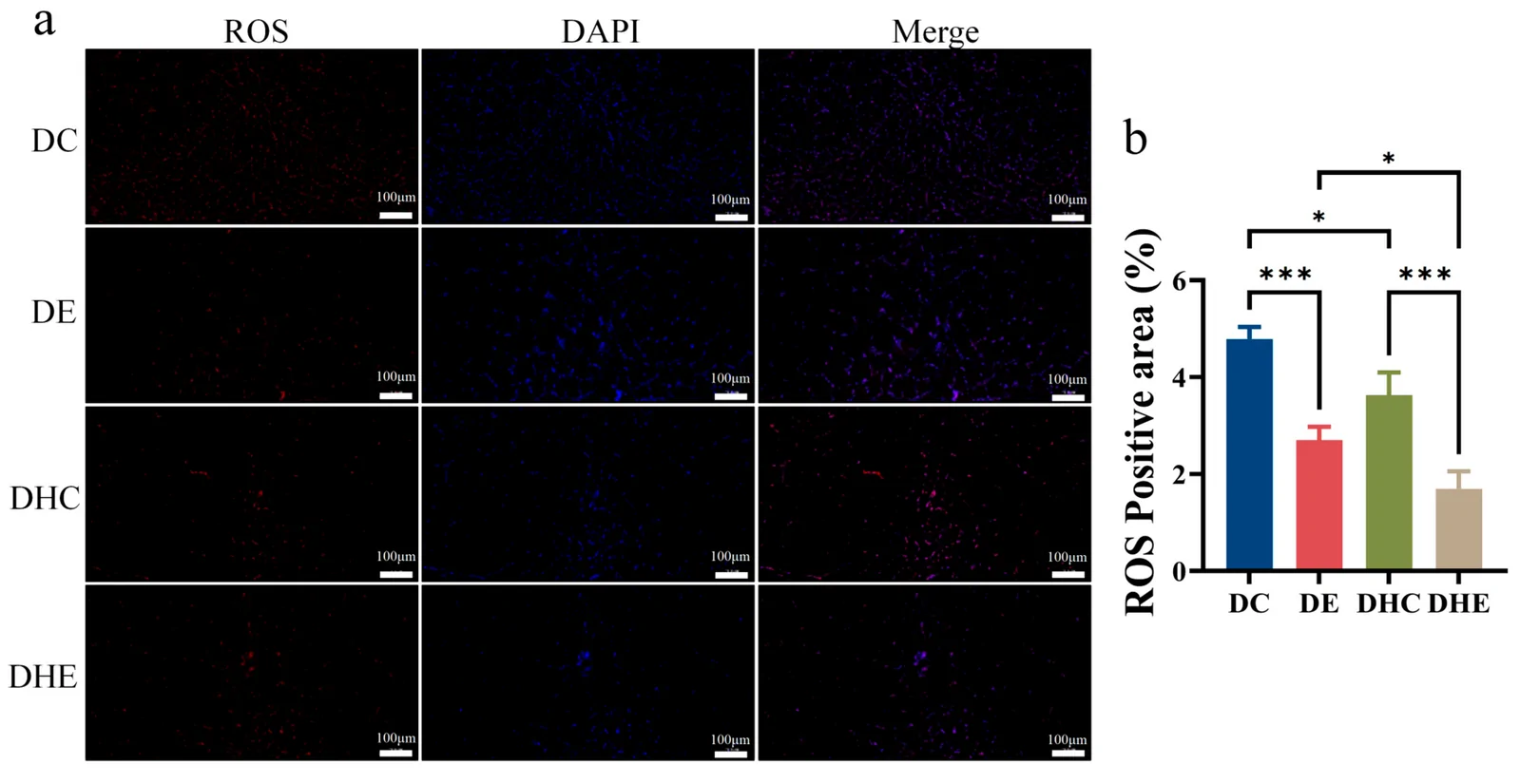
Effects of hypoxic exercise on dihydroethidium-detectable oxidative signals in skeletal muscle of zebrafish with diabetic myopathy. (**a**) Representative dihydroethidium fluorescence images of zebrafish skeletal muscle. Scale bar = 100 μm; (**b**) quantitative analysis of dihydroethidium fluorescence intensity. *n* = 3 biological replicates per group. Three non-adjacent sections were analyzed per zebrafish, with six randomly selected, non-overlapping fields of view analyzed per section; measurements were averaged to obtain one value per animal. Data are presented as mean ± SD. Statistical comparisons were performed among the DC, DE, DHC, and DHE groups. Statistical analysis was performed using two-way ANOVA followed by Tukey’s multiple comparisons test. * *p* < 0.05, *** *p* < 0.001.

Subsequently, dihydroethidium staining was performed on caudal skeletal muscle sections from zebrafish in each group (Figure 6a). Compared with the DC group, dihydroethidium fluorescence intensity was significantly decreased in the DE and DHC groups, with a more pronounced reduction observed in the DHE group (Figure 6b). These results indicate that the DHE hypoxic exercise group exhibited the greatest reduction in dihydroethidium-detectable oxidative signals among the four diabetic groups. Meanwhile, the DHE hypoxic exercise group exhibited a more pronounced reduction in ROS accumulation relative to the DE and DHC groups (Figure 6b).

### 2.7. Hypoxic Exercise Modulates Hif1a/Foxo3a-Related Signaling

**Figure 7:**
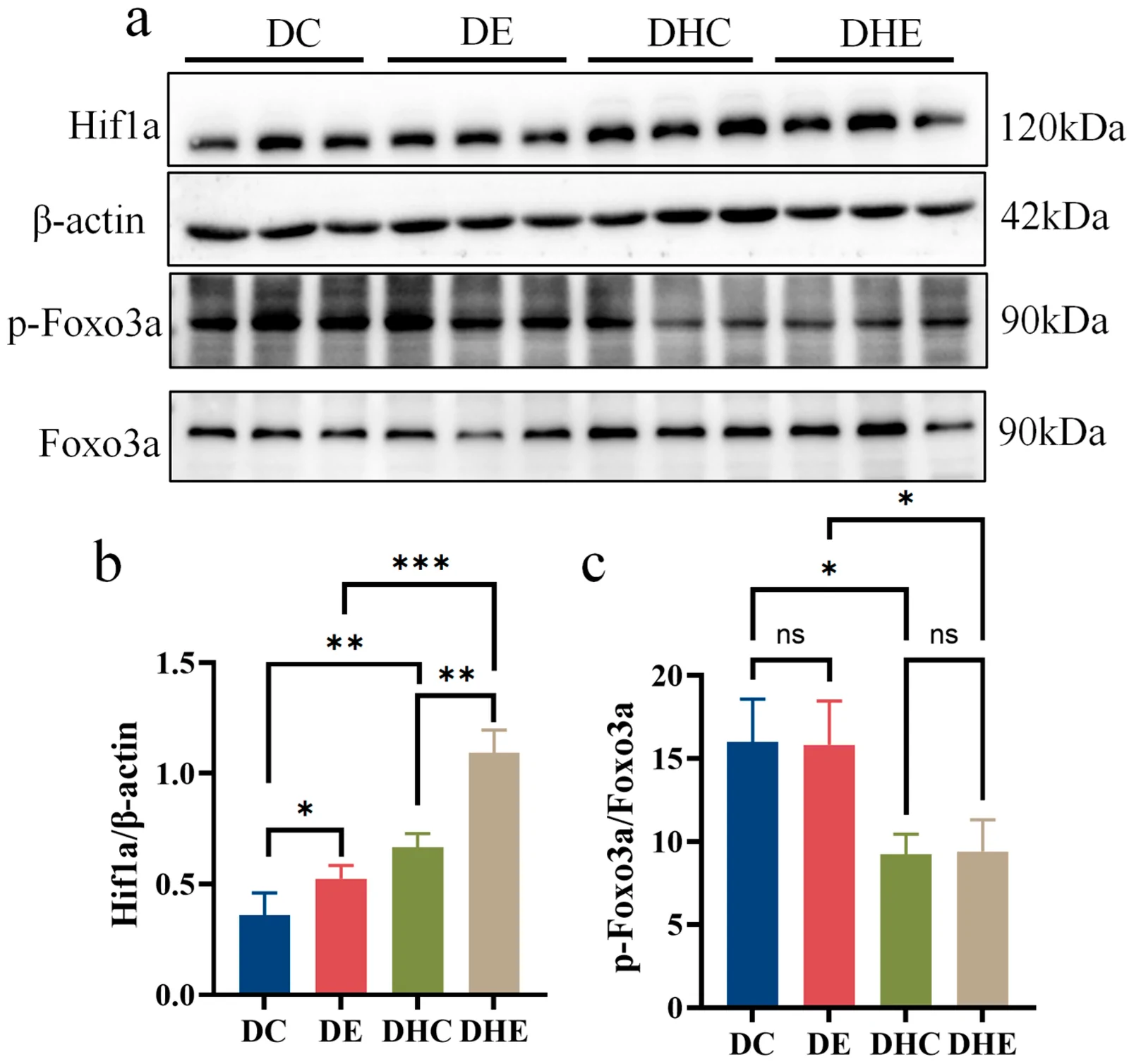
Effects of hypoxic exercise on Hif1a/Foxo3a-related signaling in skeletal muscle of zebrafish with diabetic myopathy. (**a**) Representative Western blot images of Hif1a, total Foxo3a, and p-Foxo3a; (**b**) quantitative analysis of Hif1a protein abundance; (**c**) quantitative analysis of the p-Foxo3a/Foxo3a ratio. The phosphorylated Foxo3a signal was normalized to total Foxo3a protein abundance. The p-Foxo3a antibody recognizes Foxo3a phosphorylated at Ser253. For densitometric analysis, the p-Foxo3a band at approximately 90 kDa, corresponding to the migration position of total Foxo3a, was selected for quantification. *n* = 3 biological replicates per group. Data are presented as mean ± SD. Statistical comparisons were performed among the DC, DE, DHC, and DHE groups. Statistical analysis was performed using two-way ANOVA followed by Tukey’s multiple comparisons test. ns, not significant; * *p* < 0.05, ** *p* < 0.01, *** *p* < 0.001.

To further examine molecular changes potentially associated with the effects of hypoxic exercise, we assessed Hif1a abundance and Foxo3a phosphorylation status in skeletal muscle across all groups of zebrafish. The results showed that Hif1a expression was significantly increased in the DE and DHC groups compared with the DC group, and Hif1a expression in the DHE group was markedly higher than that in the DE and DHC groups (Figure 7a,b). Meanwhile, we detected the expression and phosphorylation level of Foxo3a, a downstream target of Hif1a. The p-Foxo3a/Foxo3a ratio was significantly decreased in the DHE group compared with the DE group (Figure 7a,c). These findings indicate that hypoxic exercise is associated with increased Hif1a abundance and reduced Foxo3a Ser253 phosphorylation.

## 3. Discussion

There are multiple approaches to establish a T2DM zebrafish model, including spontaneous, chemically induced, and transgenic/knockout models [37]. The present study focused on T2DM induced by a high-sugar diet. Accordingly, referring to previous research methods, zebrafish were subjected to 8 weeks of high-sugar feeding combined with glucose immersion to successfully establish a diabetic zebrafish model [35]. In contrast to our previous study, which primarily established and characterized the diabetic myopathy zebrafish model and examined its molecular basis, the present work focuses on the therapeutic effects of hypoxic exercise and directly compares the independent and combined contributions of exercise and hypoxic exposure.

Skeletal muscle is a critical insulin-sensitive tissue and the primary site of glucose uptake in the body [38]. Together with the liver, skeletal muscle plays a central role in regulating glucose uptake and maintaining systemic glucose homeostasis [39]. Consequently, alterations in skeletal muscle health profoundly affect whole-body glucose homeostasis, further impairing muscular energy supply and recovery capacity, and ultimately leading to reduced muscle strength and endurance [40]. Skeletal muscle atrophy induced by type 2 diabetes mellitus (T2DM) is characterized by decreased muscle fiber cross-sectional area and impaired contractile function [41]. Such deterioration in skeletal muscle mass and function is defined as diabetic myopathy (DM) [6,7]. Diabetic myopathy is closely associated with reduced muscle mass and weakened muscle strength, particularly in the tissues surrounding the ankle and knee joints [42]. This pathological manifestation is closely related to ectopic lipid deposition in muscle, and the trends of fat accumulation and muscle weakness become more pronounced with aging [43]. Combined with impaired mitochondrial oxidative capacity, intramuscular lipid infiltration disrupts muscle structure and results in progressive muscle loss. In the present intervention experiment, the DC group served as the diabetic reference condition against which the effects of normoxic exercise, hypoxic exposure, and hypoxic exercise were evaluated. Accordingly, the present findings demonstrate relative differences among diabetic intervention groups rather than direct differences between diabetic and healthy zebrafish.

Exercise is a common non-pharmacological intervention that not only controls body weight but also ameliorates type 2 diabetes mellitus (T2DM) by modulating glucose metabolism. Individuals with regular physical activity exhibit a lower risk of impaired glucose tolerance compared with sedentary populations [44,45]. In recent years, increasing attention has been paid to the application of intermittent hypoxic exercise in obese populations [26]. After 3 h of normobaric hypoxia exposure, glucose metabolism decreased while lipid metabolism increased in sedentary obese males [46]. Short-term (1-week) continuous passive moderate hypoxia exposure at an altitude of 2650 m has also been shown to reduce glycated hemoglobin levels in obese individuals [47]. For obese patients with T2DM, 60 min of cycling at 90% lactate threshold under moderate hypoxic conditions (approximately 3000 m) effectively improved insulin sensitivity [27]. Further studies confirmed that 60 min of hypoxic cycling reduced fasting blood glucose and insulin concentrations in obese T2DM patients, whereas a shorter duration of 20 min failed to produce such beneficial effects [48]. These human studies provide clinical context for the metabolic effects of hypoxic exercise but cannot be directly extrapolated to zebrafish. In the present zebrafish study, hypoxic exercise produced greater improvements than normoxic exercise in several metabolic and locomotor outcomes. In the present study, hypoxic exercise, defined as endurance exercise performed under hypoxic conditions, produced greater improvements than normoxic exercise in zebrafish with diabetic myopathy. In the present design, the same absolute swimming velocity (28.8 cm/s) was deliberately applied to the DE and DHE groups to standardize the external mechanical workload. Accordingly, the DHE intervention represents the combined physiological stimulus produced by hypoxia and exercise at an identical prescribed swimming velocity. The effects of the interventions differed across individual outcomes. Normoxic exercise increased skeletal muscle CSA relative to DC, whereas DHE showed a larger CSA than DHC; however, DHE did not show an additional reduction in Trim63 abundance relative to DE or DHC. In contrast, more pronounced responses to DHE were observed for several locomotor and metabolic outcomes, indicating that the relative effects of hypoxic exercise were outcome-dependent rather than uniformly superior across all indices of diabetic myopathy, locomotor performance, and lipid accumulation compared to normoxic exercise or hypoxic exposure alone within the diabetic groups.

Mitochondria serve as the core hub of cellular energy metabolism, playing essential roles in energy regulation, redox homeostasis maintenance, and apoptosis modulation, and thus are indispensable in multiple biological processes [49]. In the present study, mitochondrial morphology and the abundance of mitochondrial quality-control-related and respiratory-chain proteins differed among the diabetic intervention groups. Hypoxic exercise was associated with more favorable mitochondrial morphology and more pronounced changes in several mitochondrial quality-control-related and respiratory-chain proteins than the comparator diabetic groups. These molecular findings characterize changes in mitochondrial-related protein abundance but do not provide direct evidence of mitochondrial respiratory function. Human studies in men with T2D have also reported exercise-associated adaptations in skeletal muscle mitochondrial quality-control-related processes [50,51]. These findings provide cross-species context but should not be interpreted as direct evidence for the mechanisms observed in zebrafish. Such abnormalities may result from the accumulation of dysfunctional and damaged mitochondria under T2DM conditions, which triggers excessive mtROS overproduction and further exacerbates skeletal muscle injury [52]. Accumulating evidence has demonstrated that increased abundance of mitophagy-related proteins protects pancreatic β-cell function and delays the progression from prediabetes to overt T2DM [53]. Normal mitochondrial function is tightly governed by three coordinated systems: mitochondrial dynamics, mitophagy, and mitochondrial biogenesis [54]. Mitophagy acts as a pivotal quality control mechanism for cellular homeostasis, and its dysfunction is closely implicated in multiple diseases. Damaged and depolarized mitochondria are selectively eliminated via mitophagy [55], a process primarily regulated by the Pink1–Parkin signaling cascade [56,57]. This pathway is critical for clearing impaired or redundant mitochondria and sustaining overall mitochondrial function [58]. Our results revealed that hypoxic exercise more markedly increased the abundance of mitophagy-related proteins, including Pink1 and Parkin, in the skeletal muscle of zebrafish with diabetic myopathy compared with normoxic exercise. Moreover, hypoxic exercise produced more pronounced changes in mitochondrial quality-control-related and respiratory-chain protein abundance, accompanied by improved mitochondrial morphology and reduced ROS accumulation in diabetic skeletal muscle. More recently, supervised resistance-based exercise has also been shown to improve skeletal muscle mitochondrial capacity in individuals with T2D, further supporting skeletal muscle mitochondria as an exercise-responsive therapeutic target [59]. It has been reported that mitophagy is enhanced with the upregulation of Hif1a expression [60]. Treatment with Hif1a agonists elevates Hif1a and Parkin protein levels in T2DM mice, thereby enhancing mitophagy and alleviating mitochondrial dysfunction [61]. Hif1 is a heterodimeric protein composed of Hif1a and Hif1b subunits. Under normoxic conditions, the Hif1a subunit is rapidly degraded through the oxygen-dependent ubiquitin–proteasome pathway [62]. In contrast, hypoxic conditions inhibit Hif1a degradation, enabling Hif1a to bind with Hif1b to form biologically active Hif1, which translocates into the nucleus to modulate the transcription of multiple target genes [63]. In addition to hypoxia, exercise alone can also gradually increase the expression and activity of Hif1a over time [64].

Previous studies have suggested functional links among Hif1a, Foxo3a, and mitophagy-related signaling under hypoxic conditions. Foxo3a knockdown has been reported to reduce Pink1 and Parkin expression and impair mitophagy [65,66], while other experimental studies have linked Hif1a signaling with Foxo3a regulation during hypoxic stress. These previous findings provide a biological context for the coordinated changes in Hif1a abundance, Foxo3a phosphorylation, and mitochondrial quality-control-related proteins observed in the present study. Collectively, hypoxic exercise was associated with increased Hif1a abundance, reduced Foxo3a Ser253 phosphorylation, altered abundance of mitochondrial quality-control-related proteins, more favorable mitochondrial morphology, and reduced oxidative signals in skeletal muscle. These coordinated changes indicate an association between hypoxic exercise and Hif1a/Foxo3a-related signaling but do not establish that this signaling pathway mediates the observed skeletal muscle effects.

Several limitations should be considered when interpreting the present findings. Although hypoxic exercise was accompanied by increased Hif1a abundance, reduced Foxo3a Ser253 phosphorylation, more favorable mitochondrial morphology, altered abundance of mitochondrial quality-control-related and respiratory-chain proteins, and reduced dihydroethidium-detectable oxidative signals, these coordinated changes establish associations rather than causal or functional relationships. Furthermore, the DHE-based ROS measurement does not differentiate mitochondrial from cytosolic contributions. Future use of mitochondria-targeted superoxide probes and oxidative-damage markers would help distinguish mitochondrial from cytosolic contributions and assess downstream oxidative injury.

In particular, the absence of pathway inhibition, genetic loss- or gain-of-function approaches, and rescue experiments precludes conclusions regarding the necessity or causal dependence of Hif1a/Foxo3a-related signaling in mediating the observed skeletal muscle effects. Likewise, changes in mitochondrial quality-control-related and respiratory-chain protein abundance do not directly demonstrate mitophagic flux or mitochondrial respiratory function. The absence of a concurrent healthy, non-diabetic control further limits assessment of the extent to which the interventions shifted diabetic abnormalities toward healthy levels. In addition, only a single hypoxic concentration, exercise intensity, and intervention duration were examined, and the study focused primarily on short-term skeletal muscle outcomes rather than whole-body metabolic and multi-organ adaptations. Future studies should therefore incorporate optimized intervention gradients, healthy controls, direct assessments of mitochondrial function and mitophagic flux, and targeted Hif1a/Foxo3a pathway manipulation, together with evaluation of Foxo3a nuclear translocation and transcriptional activity, to define the functional and causal mechanisms underlying the effects of hypoxic exercise.

Additionally, the present study was conducted exclusively in male zebrafish to control for reproductive variability, and whether these findings extend to females remains to be determined. While our protein abundance and TEM data indicate mitochondrial remodeling, they do not directly measure mitophagic flux or organelle clearance. Functional flux assays are therefore needed to determine whether the observed changes represent enhanced mitochondrial turnover.

## 4. Materials and Methods

### 4.1. Experimental Animals, T2DM Modeling, and Grouping

According to our previously established protocol [35], a total of 100 4-month-old male wild-type zebrafish from the same clutch were subjected to T2DM modeling. The model was induced by an 8-week high-glucose intervention combining a high-glucose diet with glucose immersion. Zebrafish were fed 10 mL of brine shrimp pretreated with a 50% glucose solution per feeding, three times daily. In addition, zebrafish were immersed in 5 L of glucose-containing water for 14 h each night. The glucose concentration progressively increased from 1% in week 1 to 2% in week 2, 3% in week 3, and 4% in week 4, and was maintained at 4% from weeks 5 to 8. The metabolic phenotype induced by this high-glucose protocol was validated in an independent cohort of zebrafish using fasting blood glucose, intraperitoneal glucose tolerance test (IPGTT), IPGTT area under the curve (AUC), and HbA1c measurements (Supplementary Figure S2). The animals used for metabolic model validation were independent of those subsequently assigned to the intervention groups [35]. Of the 100 zebrafish subjected to T2DM modeling for the intervention experiment, 17 died during the modeling period and 3 were excluded before randomization because of markedly abnormal body length or body weight. The remaining 80 eligible T2DM zebrafish were randomly assigned to four groups (*n* = 20 per group): the T2DM normoxia control group (DC), T2DM normoxia exercise group (DE), T2DM hypoxia control group (DHC), and T2DM hypoxia exercise group (DHE). In the present experiment, model establishment was reconfirmed by impaired glucose tolerance using IPGTT before randomization. 

The high-glucose induction protocol was metabolically validated in an independent cohort of zebrafish by fasting blood glucose, IPGTT, IPGTT AUC, and HbA1c measurements (Supplementary Figure S2). The animals used for model validation were not included in the subsequent exercise-intervention groups. The overall experimental workflow is summarized in Supplementary Figure S1.

All experimental fish were maintained at 28.0 ± 0.5 °C under a 14 h:10 h light-dark cycle with oxygen-enriched recirculating water. Fish were fed brine shrimp three times daily at 8:00 a.m., 12:00 noon, and 6:00 p.m. All animal care and experimental procedures were performed in compliance with the Laboratory Animal Management and Use Guidelines of Hunan Normal University, with ethical approval number 2024-458.

### 4.2. Zebrafish Exercise Protocol

Subsequently, the critical swimming speed (Ucrit) of zebrafish was determined under normoxic conditions according to our previously established protocol [35], with an average value of 41.15 cm/s. Thereafter, 70% Ucrit (28.8 cm/s) was used as the same absolute swimming velocity for both the DE and DHE groups to standardize the external mechanical workload. The DC and DE groups underwent sedentary control and normoxic exercise, respectively, at 100% air saturation. The DHC group received intermittent hypoxic exposure without exercise, whereas the DHE group performed intermittent hypoxic exercise at 66% air saturation. Hypoxic exposure was restricted to the daily 2 h intervention sessions and was controlled using a Loligo System Oxygen Analyzer (OX10000, Loligo Systems, Seeland, Denmark) and Solenoid Valve (AC10050, Loligo Systems, Seeland, Denmark) During each hypoxic session, dissolved oxygen was continuously monitored and maintained at 66% air saturation. Water temperature was maintained at 28.0 ± 0.5 °C, and pH was maintained at 7.0–7.5. The training chamber had a volume of 1 L and contained 20 zebrafish (20 fish/L). For the exercise groups, water velocity was maintained at 28.8 cm/s. The system required approximately 1 min to reach 66% air saturation before each hypoxic intervention. All interventions were performed for 2 h/day, 5 days/week, for a total of 5 weeks. No exercise was imposed in the sedentary control groups.

### 4.3. Intraperitoneal Glucose Tolerance Test (IPGTT)

Zebrafish were subjected to a 24 h fasting period before the test. On the day of the experiment, the required buffer solution was prepared, and 5 g of glucose was dissolved in 10 mL of buffer. Fish were weighed and labeled individually, followed by intraperitoneal injection of glucose at a dosage of 0.5 mg/g body weight. Blood glucose levels were then measured at fasting baseline, 30 min, 90 min, and 180 min post-injection using a micro blood glucose meter (Yuwell, Wenzhou, China).

### 4.4. Zebrafish Behavioral Testing

A high-speed camera was installed and positioned to fully capture the glass petri dishes placed on an illuminated plate. Camera exposure and focal length were adjusted to ensure clear imaging. After launching the Ueye software(version 4.96.1), one zebrafish per test subject was placed into each of four glass petri dishes and allowed to acclimate undisturbed for 10 min. A new file was created in Ueye, and spontaneous behavior in the open-field petri dish was recorded for 1 min. Recorded videos were then imported into Lolitrack software (version 4.2.1, Loligo Systems, Seeland, Denmark). The swimming arena and initial position of each fish were defined, followed by analyses of total distance traveled, average velocity, acceleration, and trajectory mapping.

### 4.5. Sample Collection

On the day following the completion of exercise training, experimental fish were euthanized via ice-water immersion. Body mass was measured using an electronic balance, and blood glucose levels were determined using a blood glucose meter, with surplus blood collected for subsequent analyses.

Skeletal muscle was dissected according to standard zebrafish anatomical procedures. Briefly, skeletal muscle from the posterior region of the dorsal fin to the anterior margin of the caudal fin was carefully isolated using a scalpel, with care taken to maintain tissue integrity. Samples intended for Western blot analysis were immediately frozen in liquid nitrogen and then stored at −80 °C until use.

Tissues for hematoxylin and eosin (HE) staining were fixed in 4% paraformaldehyde (0.1 M, pH 7.4) for a minimum of 12 h. Samples for Oil Red O and dihydroethidium staining were snap-frozen in liquid nitrogen and stored at −80 °C. Dihydroethidium staining was performed on fresh-frozen skeletal muscle sections prepared without chemical fixation. For all histological and fluorescence measurements, tissue sections from the same anatomical region of three randomly selected zebrafish per group were used for quantitative analysis. Three non-adjacent sections were selected from each zebrafish, and six randomly selected, non-overlapping fields of view were analyzed per section. Skeletal muscle tissues prepared for transmission electron microscopy (TEM) to assess mitochondrial morphology were preserved in an electron microscopy fixative and stored at 4 °C. The TEM samples were observed under a JEM1400 transmission electron microscope (JEOL Ltd., JEM1400, Shimoji City, Japan), and images were recorded using a Morada G3 digital camera (EMSIS GmbH, Minster, Germany).

For all histological and fluorescence measurements, tissue sections from the same anatomical region of three randomly selected zebrafish per group were used for quantitative analysis. Three non-adjacent sections were selected from each zebrafish, and six randomly selected, non-overlapping fields of view were analyzed per section. All images were acquired and quantified under identical conditions by investigators blinded to the experimental groups to minimize potential bias.

### 4.6. Assessment of Mitochondrial Damage

Mitochondrial ultrastructural damage observed under transmission electron microscopy (TEM) was quantified via Flameng’s scoring system, following the analytical protocol detailed in reference [66]. For each group, three zebrafish were examined, and five random visual areas were captured at a unified magnification of 7000× for every experimental zebrafish; roughly 20 mitochondria within each captured field were picked out for subsequent quantitative evaluation. Mitochondrial lesions were stratified into five grades ranging from 0 to 4 based on the severity of structural impairment:

Grade 0: intact and normal mitochondrial ultrastructure;

Grade 1: intact cristae and matrix compartments, with no granular matrix deposits visible;

Grade 2: disappearance of matrix granules and matrix rarefaction, while mitochondrial cristae remain intact;

Grade 3: fractured cristae accompanied by matrix clearing and matrix condensation;

Grade 4: complete cristae breakdown plus ruptured mitochondrial outer and inner membranes.

### 4.7. Western Blot Analysis

Steel beads were added to 1.5 mL EP tubes and labeled accordingly. RIPA lysis buffer was freshly prepared at a ratio of RIPA:PMSF:phosphatase-inhibitor cocktail = 100:1:1. Tissues were weighed and transferred into EP tubes with recorded weights, followed by the addition of RIPA lysis buffer at a ratio of 1 mg tissue to 10 μL buffer. The tubes were placed in a pre-cooled high-throughput tissue homogenizer and homogenized at 60 Hz for 60 s per cycle, repeated three times to ensure complete homogenization. Homogenates were then incubated for lysis at 4 °C for 30 min, followed by centrifugation at 12,000 rpm at 4 °C for 15 min. The supernatant (crude protein extract) was carefully transferred to new EP tubes to avoid contamination with debris or precipitates. Protein concentration was quantified using the BCA assay. The remaining supernatant was mixed with 5× SDS loading buffer at a ratio of 4:1, followed by denaturation in boiling water for 10 min prior to subsequent analysis.

Protein samples (30 μg per lane) were loaded onto SDS–PAGE gels. Electrophoresis was performed at a constant voltage of 80 V for the stacking gel and 120 V for the separating gel. Upon completion, the gel was carefully excised and immersed in pre-chilled transfer buffer (Servicebio, Wuhan, China). The gel and PVDF membrane were assembled in a transfer cassette, and electrophoretic transfer was conducted at a constant current of 400 mA for 30 min. Membranes were then blocked with 5% non-fat milk at room temperature for 2 h. After blocking, milk was discarded and membranes were rinsed with TBST. Primary antibodies were diluted with TBST according to the manufacturers’ instructions, and membranes were incubated with diluted primary antibodies in antibody incubation boxes overnight at 4 °C. After incubation, primary antibodies were recovered, and membranes were washed three times with TBST on a shaker at high speed for 10 min each. Corresponding secondary antibodies were then applied and incubated at room temperature for 2 h. Subsequently, the secondary antibody solution was removed, and membranes were washed three times with TBST for 10 min each. For visualization and quantification, ECL working solution was prepared by mixing reagent A and reagent B at 1:1, and PVDF membranes were incubated in the solution. All β-actin loading controls were obtained from the same membranes used for target protein detection. Signals were captured using a chemiluminescence imaging system (Tanon 5200 multi, Shanghai, China), and band gray values were analyzed using ImageJ software (version 1.8.0.332).

The p-Foxo3a antibody (ImmunoWay Biotechnology, YM8032, Calif., Cal, [country]) recognizes Foxo3a phosphorylated at Ser253. For densitometric analysis, the band migrating at approximately 90 kDa, consistent with the expected molecular weight of Foxo3a and the corresponding total Foxo3a band, was selected for quantification. Other bands were excluded from the analysis.

Detailed information on the primary antibodies used for Western blotting, including the target protein, source, catalog number, and dilution factor, is provided in Supplementary Table S2.

### 4.8. Data Analysis

Statistical analyses of all experimental data were performed using SPSS 22.0 software. All data were presented as mean ± standard deviation (SD). Two-way analysis of variance (two-way ANOVA) was applied to evaluate the two experimental factors in the hypoxic training model, namely oxygen concentration and exercise training (see Supplementary Table S1 for details). The main effect of each factor and the interaction effect between oxygen concentration and exercise training were assessed. Subsequently, one-way ANOVA followed by Tukey’s multiple-comparisons test was used for post hoc comparisons among the four groups. was conducted to identify intergroup differences among the four experimental groups. GraphPad Prism software 8.0 (GraphPad Software, San Diego, CA, USA) was used to generate all graphs. *p* < 0.05 was considered statistically significant.

For histological, fluorescence, and TEM analyses, measurements from multiple sections or fields of view within the same zebrafish were averaged to obtain one value per animal, and the individual zebrafish was used as the biological and statistical unit. Technical measurements were not treated as independent replicates.

For Western blot analysis, *n* represents independent biological samples obtained from individual zebrafish.

## 5. Conclusions

This study demonstrated that hypoxic exercise effectively ameliorates diabetic myopathy in zebrafish, accompanied by modulation of Hif1a/Foxo3a-related signaling and improved mitochondrial quality control.

## Data Availability

The data presented in this study are available from the corresponding author upon reasonable request, as they are part of an ongoing study and have not yet been publicly released.

## Supplementary Materials

The following supporting information can be downloaded at: https://www.mdpi.com/article/10.3390/ijms27188034/s1.
