# Supplementary material for: Hypoxic Exercise Alleviates Diabetic Skeletal Myopathy in Zebrafish in Association with Hif1a/Foxo3a Signaling and Mitochondrial Quality Control
Source: Int J Mol Sci. 2026 Sep 9;27(18):8034. doi: 10.3390/ijms27188034 (PMC13606683; doi:10.3390/ijms27188034)
Supplement: Supplementary file 1 [file ijms-27-08034-s001.zip › ijms-4478570-supplementary.pdf]

Supplementary Figure S1. Experimental workflow of the study.

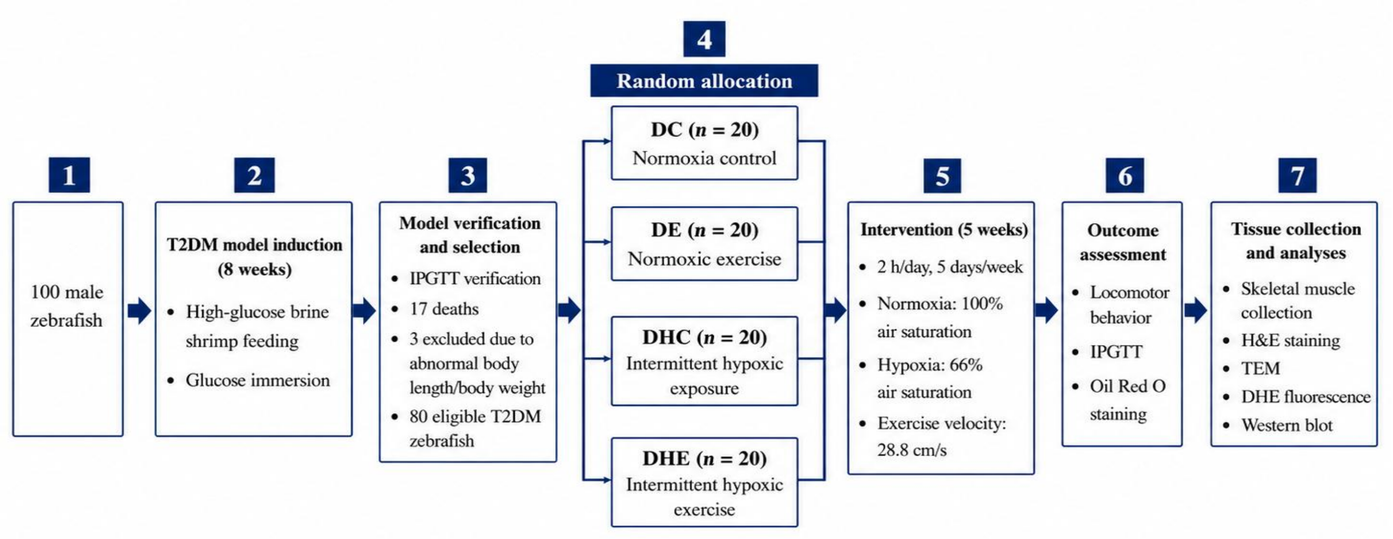

Supplementary Figure S1. Experimental workflow of the study. Male zebrafish were subjected to 8 weeks of T2DM model induction, followed by model verification and random allocation into four groups. After a 5-week intervention period, behavioral and metabolic assessments were performed, followed by tissue collection and histological and molecular analyses.

Supplementary Figure S2. Metabolic validation of the high-glucose-induced T2DM zebrafish model.

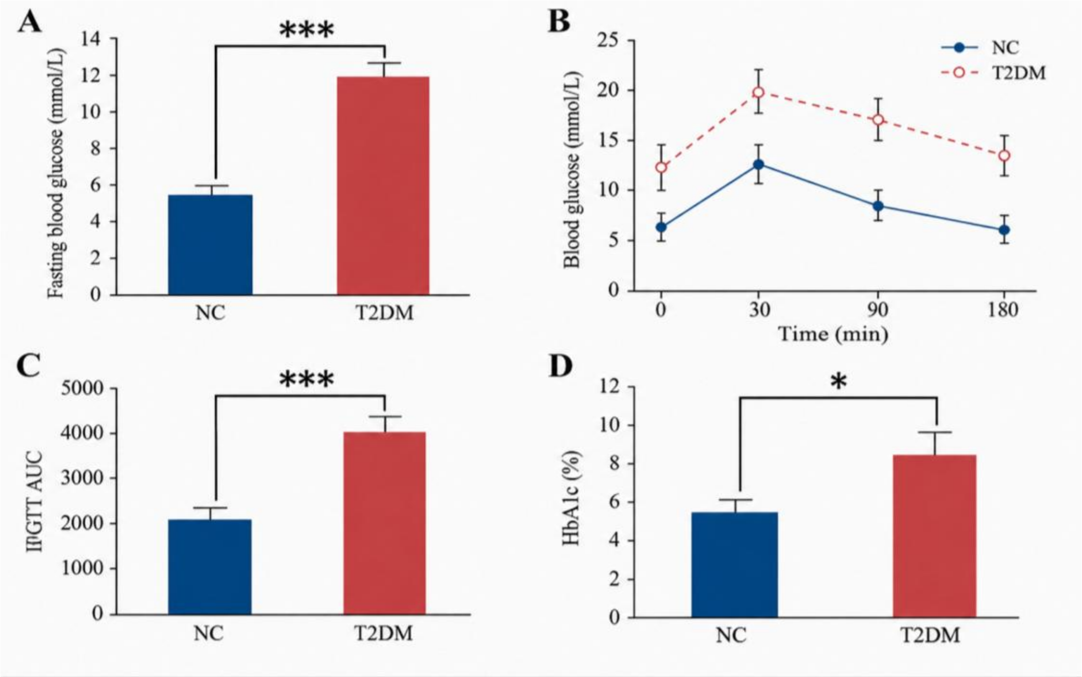

Supplementary Figure S2. Metabolic validation of the high-glucose-induced T2DM zebrafish model. (A) Fasting blood glucose levels in NC and T2DM zebrafish. (B) Intraperitoneal glucose tolerance test (IPGTT) curves. (C) Area under the curve (AUC) of the IPGTT. (D) HbA1c levels. Compared with the NC group, T2DM zebrafish exhibited elevated fasting blood glucose, impaired glucose tolerance, increased IPGTT AUC, and elevated HbA1c levels. Data are presented as mean ± SD; n = 6 biological replicates per group. Statistical analysis was performed using an unpaired t-test. *$p < 0.05$, ***$p < 0.001$ vs. NC.

Supplementary Figure S3. Terminal fasting blood glucose and body weight of T2DM zebrafish following the intervention.

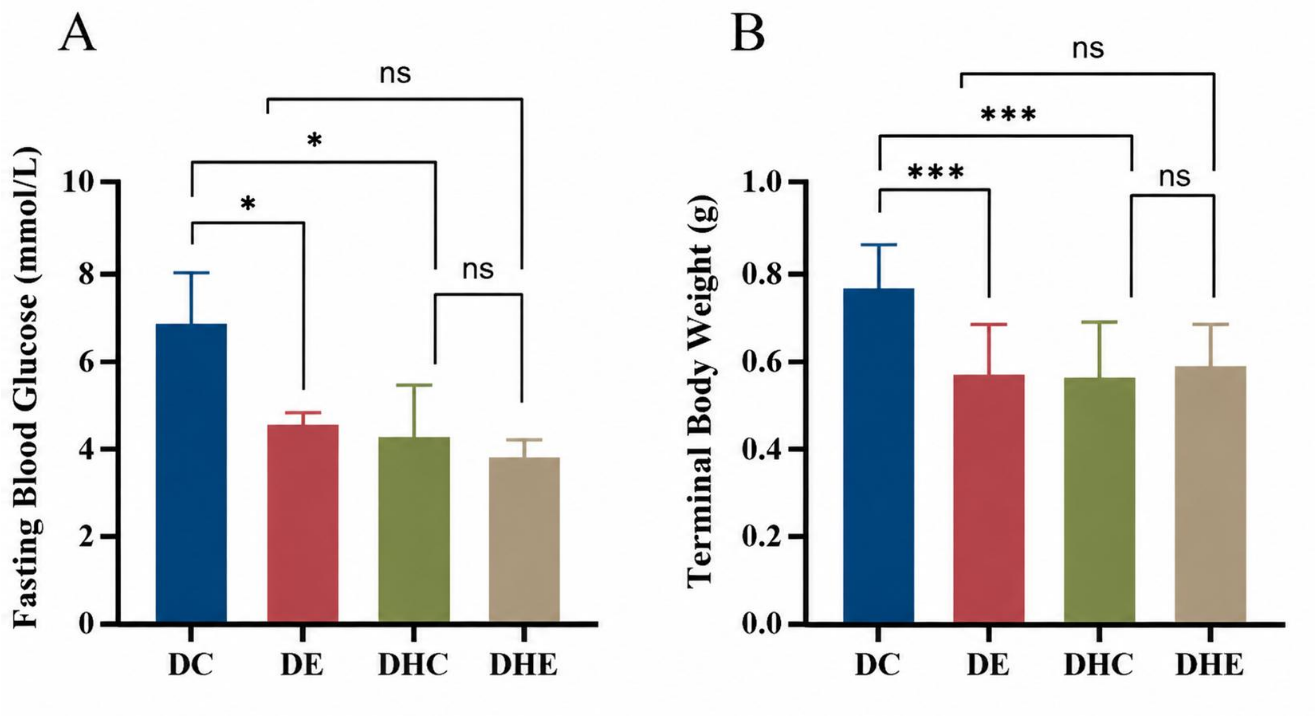

Supplementary Figure S3. Terminal fasting blood glucose and body weight of T2DM zebrafish following the intervention. (A) Fasting blood glucose levels(n = 6 per group). (B) Terminal body weight. Data are presented as mean ± SD (n = 20 per group). DC, T2DM normoxia control group; DE, T2DM normoxia exercise group; DHC, T2DM hypoxia control group; DHE, T2DM hypoxia exercise group. ns, not significant; Statistical analysis was performed using one-way ANOVA followed by Tukey's multiple comparisons test. *$p < 0.05$; ***$p < 0.001$.

Table S1. Results of two-way ANOVA

Variable	Factor	F	p-value
IPGTT AUC	Oxygen concentration	51.384	<0.001
	Exercise intervention	39.574	<0.001
	Oxygen concentration × Exercise interaction	0.125	0.733
Oil Red O positive area (%)	Oxygen concentration	22.048	0.002
	Exercise intervention	64.487	<0.001
	Oxygen concentration × Exercise interaction	0.151	0.707
CSA (μm²)	Oxygen concentration	4.621	0.032
	Exercise intervention	77.655	<0.001
	Oxygen concentration × Exercise interaction	1.697	0.193
Trim63/ β -actin	Oxygen concentration	13.735	0.006
	Exercise intervention	19.576	0.002
	Oxygen concentration × Exercise interaction	5.267	0.051
Ucrit (cm/s)	Oxygen concentration	36.779	<0.001
	Exercise intervention	70.406	<0.001
	Oxygen concentration × Exercise interaction	0.153	0.706
Ucrit-r (BL/s)	Oxygen concentration	47.098	<0.001
	Exercise intervention	68.276	<0.001
	Oxygen concentration × Exercise interaction	0.016	0.902
Avg. velocity (cm/s)	Oxygen concentration	32.855	<0.001
	Exercise intervention	31.092	<0.001
	Oxygen concentration × Exercise interaction	1.656	0.222
Avg. Acceleration (cm/s²)	Oxygen concentration	71.518	<0.001
	Exercise intervention	97.584	<0.001
	Oxygen concentration × Exercise interaction	16.597	0.002
Active time (%)	Oxygen concentration	183.809	<0.001
	Exercise intervention	211.580	<0.001
	Oxygen concentration × Exercise interaction	67.143	<0.001
Distance moved (cm)	Oxygen concentration	186.823	<0.001
	Exercise intervention	214.021	<0.001
	Oxygen concentration × Exercise interaction	1.638	0.225
Flameng's score	Oxygen concentration	46.286	<0.001
	Exercise intervention	28.000	<0.001
	Oxygen concentration × Exercise interaction	0.571	0.457
Tfam/ β -actin	Oxygen concentration	29.509	<0.001

	Exercise intervention	4.098	0.078
	Oxygen concentration × Exercise interaction	0.385	0.552
Drp1/β-actin	Oxygen concentration	7.707	0.024
	Exercise intervention	6.077	0.039
	Oxygen concentration × Exercise interaction	0.987	0.349
Opa1/β-actin	Oxygen concentration	95.030	<0.001
	Exercise intervention	24.761	0.001
	Oxygen concentration × Exercise interaction	0.023	0.883
Mfn2/β-actin	Oxygen concentration	21.063	0.002
	Exercise intervention	13.625	0.006
	Oxygen concentration × Exercise interaction	6.263	0.037
Pink1/β-actin	Oxygen concentration	33.973	<0.001
	Exercise intervention	4.413	0.069
	Oxygen concentration × Exercise interaction	0.345	0.573
Parkin/β-actin	Oxygen concentration	22.483	0.001
	Exercise intervention	14.156	0.006
	Oxygen concentration × Exercise interaction	1.943	0.201
Ndufb8/β-actin	Oxygen concentration	4.891	0.058
	Exercise intervention	3.790	0.087
	Oxygen concentration × Exercise interaction	0.140	0.718
Sdhb/β-actin	Oxygen concentration	49.741	<0.001
	Exercise intervention	30.755	<0.001
	Oxygen concentration × Exercise interaction	0.820	0.392
Uqcrc2/β-actin	Oxygen concentration	20.369	0.002
	Exercise intervention	3.894	0.084
	Oxygen concentration × Exercise interaction	0.016	0.901
Atp5a/β-actin	Oxygen concentration	42.093	<0.001
	Exercise intervention	11.631	0.009
	Oxygen concentration × Exercise interaction	2.584	0.147
ROS positive area (%)	Oxygen concentration	28.715	<0.001
	Exercise intervention	98.753	<0.001
	Oxygen concentration × Exercise interaction	0.148	0.711
Hif1a/β-actin	Oxygen concentration	82.147	<0.001
	Exercise intervention	37.348	<0.001
	Oxygen concentration × Exercise interaction	7.273	0.027
p-Foxo3a/Foxo3a	Oxygen concentration	28.022	<0.001
	Exercise intervention	0.000	0.995
	Oxygen concentration × Exercise interaction	0.019	0.893

Table S2. Primary antibodies used for Western blotting

Target Protein	Source	Catalog Number	Dilution Factor
Trim63	Cell Signaling Technology, USA	33973	1:1000
β -actin	Bioss, China	bsm-63325R	1:5000
Tfam	Proteintech, USA	22586-1-AP	1:15000
Drp1	Proteintech, USA	12957-1-AP	1:5000
Opa1	Proteintech, USA	27733-1-AP	1:5000
Mfn2	Proteintech, USA	12186-1-AP	1:5000
Pink1	Proteintech, USA	23274-1-AP	1:2000
Parkin	Boster Biological Technology, USA	AOCD-16	1:1000
Ndufb8	Proteintech, USA	14794-1-AP	1:5000
Sdhb	Proteintech, USA	10620-1-AP	1:15000
Uqcrc2	Proteintech, USA	14742-1-AP	1:5000
Atp5a	Proteintech, USA	14676-1-AP	1:15000
Foxo3a	Proteintech, USA	10849-1-AP	1:5000
Hif1a	GeneTex, USA	GTX127309	1:1000
p-Foxo3a	ImmunoWay, USA	YM8032	1:2000

Note：The p-Foxo3a antibody recognizes Foxo3a phosphorylated at Ser253. For densitometric analysis, the band at approximately 90 kDa corresponding to the migration position of total Foxo3a was selected for quantification. Additional bands were excluded from the analysis.
